# Design of Targeted Flurbiprofen Biomimetic Nanoparticles for Management of Arthritis: In Vitro and In Vivo Appraisal

**DOI:** 10.3390/pharmaceutics14010140

**Published:** 2022-01-07

**Authors:** Hagar I. Mohamed, Amal H. El-Kamel, Ghada O. Hammad, Lamia A. Heikal

**Affiliations:** 1 Department of Pharmaceutics, Faculty of Pharmacy, Alexandria University, Alexandria 21521, Egypt; hagarelsherif7171@gmail.com (H.I.M.); lamia.heikal@alexu.edu.eg (L.A.H.); 2 Department of Pharmacology & Therapeutics, Faculty of Pharmacy, Pharos University in Alexandria, Alexandria 21526, Egypt; ghada.osama@pua.edu.eg

**Keywords:** arthritis, flurbiprofen, nanoparticles, intra-articular, hyaluronic acid, active targeting

## Abstract

Flurbiprofen (FLUR) is a potent non-steroidal anti-inflammatory drug used for the management of arthritis. Unfortunately, its therapeutic effect is limited by its rapid clearance from the joints following intra-articular injection. To improve its therapeutic efficacy, hyaluronic acid-coated bovine serum albumin nanoparticles (HA-BSA NPs) were formulated and loaded with FLUR to achieve active drug targeting. NPs were prepared by a modified nano-emulsification technique and their HA coating was proven via turbidimetric assay. Physicochemical characterization of the selected HA-BSA NPs revealed entrapment efficiency of 90.12 ± 1.06%, particle size of 257.12 ± 2.54 nm, PDI of 0.25 ± 0.01, and zeta potential of −48 ± 3 mv. The selected formulation showed in-vitro extended-release profile up to 6 days. In-vivo studies on adjuvant-induced arthritis rat model exhibited a significant reduction in joint swelling after intra-articular administration of FLUR-loaded HA-BSA NPs. Additionally, there was a significant reduction in CRP level in blood as well as TNF-α, and IL-6 levels in serum and joint tissues. Immunohistochemical study indicated a significant decrease in iNOS level in joint tissues. Histopathological analysis confirmed the safety of FLUR-loaded HA-BSA NPs. Thus, our results reveal that FLUR loaded HA-BSA NPs have a promising therapeutic effect in the management of arthritis.

## 1. Introduction

Arthritis is a complicated chronic disease characterized by severe inflammation of joints and irreversible damage of bone and cartilage. This results in complete disability of patients to practice their daily life within a few years of disease progression [1]. It has been reported that almost 1% of the world’s population is affected by arthritis [2]. Despite not being age-related, arthritis develops most commonly in elderly people [3]. Natural environmental fluctuation such as barometric pressure and temperature play an important role in intensifying the pain induced by arthritis [4]. There are different types of arthritis, and osteoarthritis and rheumatoid arthritis are the main types. The pathogenesis of arthritis involves different mechanisms of the immune system leading to multiple immunological responses that ultimately result in joint damage [5]. Interaction between various immune cells (B-cells, T-cells, macrophages, and synoviocytes) and angiogenesis are responsible for the inflammation occurring in the joints. Activation of B-cells and T-cells leads to over-production of inflammatory cytokines and chemokines such as tumor necrosis factor (TNF-α) and interleukin-6 (IL-6) which result in further B-cells, T-cells, and macrophages interaction [6]. 

The available clinical treatments for the management of arthritis involve non-steroidal anti-inflammatory drugs (NSAIDs), glucocorticoids (GC), and disease-modifying anti-rheumatic drugs (DMARDs) [7]. GC such as dexamethasone, prednisolone can rapidly inhibit inflammation but exhibit severe adverse effects (Immunosuppression, Osteoporosis, Hyperglycemia, and Hypertension) which limit their long-term use [8]. DMARDs can suppress the acute phase of arthritis disease and inhibit joint damage [9]. They include synthetic agents such as methotrexate (the first-line drug for arthritis) and different biological agents (cytokines antagonists, B-cells depleting agents, T-cells signaling inhibitor, and kinase enzyme inhibitors) [10]. The mechanism of action of these drugs is not common and every drug has its side effects, but the most common side effects are gastrointestinal dysfunction, liver disorders, kidney dysfunction, and myelosuppression [11]. 

NSAIDs have a vital role in the management of arthritis via inhibition of cyclooxygenase enzyme (COX) which leads to suppression of prostaglandin production reducing pain, inflammation, and swelling [12]. Despite their side effects such as renal dysfunction, cardiovascular disorder, and gastrointestinal problems [8], NSAIDs are considered the most common and widely used drugs for arthritis owing to their analgesic and anti-inflammatory effects [11]. Flurbiprofen (FLUR) is the most potent NSAID that is widely used for the management of arthritis (both osteoarthritis and rheumatoid arthritis). It acts by inhibiting the COX-2 enzyme resulting in the suppression of the production of prostaglandin and consequently reducing inflammation and pain [13]. According to the Biopharmaceutical classification system, FLUR belongs to class II, which is characterized by poor water solubility and high permeability [14]. It has a short half-life (around four hours) thus, it is given orally in different doses (50–75 mg) three or four times daily to achieve the required therapeutic concentration [15]. Due to the different adverse effects of FLUR such as GIT irritation, bleeding after oral administration and the low drug concentration reaching the synovial fluid due to systemic exposure, the intra-articular delivery route of FLUR is highly recommended as an alternative for other routes to overcome these problems [15]. Intra-articular drug delivery is a commonly used route for different types of arthritis disease as it allows direct delivery of drugs into joints, avoids systemic exposure, improves the bioavailability of drugs at the disease site, and reduces side effects [16]. However, the rapid clearance of drugs from joints (6 h) is the main problem that limits the therapeutic effect of the intra-articular route [17].

Currently available intra-articular treatment is limited. For example, corticosteroids intra-articular injection is used to reduce pain and inflammation but still has a short half-life and cannot prevent the progression of the disease [18,19]. U.S. Food & Drug Administration (FDA) approved a hyaluronic acid (HA) intra-articular injection formulation as a viscosupplementation formulation that is used for the treatment of arthritis, but it cannot be used as a substitute for joint replacement [20]. Although intra-articular injection offers direct access of drugs into inflamed joints, patients remain suffering from repeated needling which increases the risk of infection [21].

The application of nanomedicine has several advantages in improving the therapeutic outcome of drugs in the management of arthritis. It helps in retaining the drug within inflamed joints with an extended-release effect. This could help in overcoming the rapid clearance of drugs from joints after intra-articular injection [22]. In addition, nanotherapeutics help in achieving more precise treatment via passive and active targeting strategies improving the therapeutic effect of drugs and decreasing their toxicity [23]. 

Owing to the advantages of nanomedicine, various types of nano-based delivery systems have been developed [24]. In polymer-drug conjugate (PDC) delivery system a backbone of different polymers such as polyethylene glycol (PEG), polyvinylpyrrolidone (PVP), hydroxypropyl-meth-acrylamide (HPMA), and natural polymers as Hyaluronic acid (HA) attached to therapeutic agents or targeting moieties have been reported [25]. Unlike PDC, nanoparticulate systems have the advantages that they can encapsulate and entrap drugs without any modification [25]. The large surface area of NPs allows modification of drug properties such as solubility, half-life, diffusivity, and drug release. They also allow drug targeting to the specific site via active or passive targeting [26]. To minimize the uptake of nanoparticles (NPs) by macrophages of the reticuloendothelial system, their size should range between 20 and 300 nm which can permit passive targeting to inflamed tissues via leaky vasculature; an important characteristic of inflamed tissue [27,28].

Albumin is the most abundant plasma protein produced by hepatocytes. It is non-toxic, biodegradable, biocompatible, and widely used as a drug carrier [29]. Additionally, albumin is a good candidate to be used as a targeting ligand in arthritis that can be selectively oriented to inflamed joints suffering from hypoalbuminemia [30]. Carvacrol albumin NPs were developed to encapsulate carvacrol where its immunomodulatory effect in the arthritis rat model was investigated. Results showed a significant decrease in severity score, erythrocyte sedimentation rate, nitric oxide production, and IL-17 expression compared to the untreated group [31].

Active targeting techniques for inflammatory synovial regions can be used to provide targeted drug distribution and improve drug therapeutic efficacy where an appropriate ligand for drug delivery is chosen that can specifically bind to certain cells at the target spot [32]. In cases of inflammation, CD44 receptors are overexpressed compared to normal cells [33]. As a result, compounds that can specifically bind to CD44 receptors, such as HA, can be employed as a possible targeting ligand [34]. HA is a natural polysaccharide that is frequently used for drug delivery in arthritis disease. It is an important component of synovial fluid and improves the condition of joints [35]. NPs based on HA and albumin are good drug delivery nanoplatforms for the management of arthritis owing to their stability, feasibility, and biocompatibility [36]. HA-coated albumin NPs loaded with brucine as an anti-inflammatory drug were prepared for intra-articular injection in rats which were capable to reside in the articular cavity of rats for more than 14 days [37].

Consequently, the main aim of this study was to develop an intra-articular FLUR biomimetic nano-formulation for the management of arthritis which can target inflamed joints via passive and active targeting. The targeting of joints could reduce systemic side effects of FLUR, increase the effectiveness of the drug and avoid rapid clearance of the drug from joints. To achieve joint targeting, HA-coated albumin-based NPs loaded with FLUR were prepared, injected intra-articularly into arthritis-induced rats and the anti-arthritic effect of this formulation was evaluated pharmacologically, histologically, and by molecular techniques. Up to our knowledge, our study is considered the first to formulate FLUR-loaded albumin NPs coated with HA to target the inflamed joints in arthritis.

## 2. Materials and Methods

### 2.1. Materials

Flurbiprofen (FLUR), Hyaluronic acid (HA), Bovine serum albumin (BSA), Cetyltrimethylammonium bromide (CTAB), and Complete Freund’s adjuvant (CFA) were purchased from Sigma-Aldrich (St. Louis, MO, USA). Chloroform, Methanol, and Citric acid were purchased from Al-Gomhureya Co. (Cairo, Egypt). All other reagents and chemicals were of analytical grade.

### 2.2. Preparation of Bovine Serum Albumin Nanoparticles (BSA NPs)

BSA NPs were prepared by a modified nano-emulsification technique [38]. Briefly, BSA (30, 50, and 60 mg) was dissolved in 2 mL deionized water, and the pH of the solution was adjusted between 5.6 and 5.9 using 10% *w*/*v* citric acid. To prepare FLUR-loaded BSA NPs, FLUR (15, 25, and 40 mg) was dispersed separately in 0.5 mL of chloroform then added dropwise to the prepared BSA solution under vigorous stirring (800 rpm) via a magnetic stirrer (IKA Labortechnik, Staufen, Germany) for at least an hour. The mixture was then homogenized for 5 min at 15,000 rpm in an ice bath using a high shear homogenizer (Ultra Turrax; IKA Labortechnik, Staufen, Germany). NPs were formed upon exposure to high shear stress. The NPs suspension was centrifuged at 13,000 rpm, 4° for 30 min (Model 3K-30; Sigma Laborzentrifugen GmbH, Osterode, Germany), the supernatant was then removed, and the pellet of NPs was re-dispersed in 0.9% *w*/*v* normal saline.

### 2.3. Preparation of Hyaluronic Acid-Coated BSA NPs (HA-BSA NPs)

HA-BSA NPs were prepared based on adsorption of negatively charged HA onto the NPs surface [37]. The pH of BSA NPs dispersion was first adjusted to 6.5 using 1 M NaOH. HA colloidal solution (2 mg/mL) was added dropwise into the NP dispersion in different volumes (0.5, 1, or 2 mL) under vigorous stirring at 800 rpm via magnetic stirrer (IKA Labortechnik, Staufen, Germany) for 2 h at 37 °C to ensure complete coating of the NPs. The resulting HA-BSA NPs were stored at 4 °C overnight for stabilization.

### 2.4. Determination of Hyaluronic Acid Content

The amount of HA coated on BSA NPs was determined by a turbidimetric method which is based on the formation of turbidity between HA and CTAB reagent [39]. First, the calibration curve of HA was prepared where 2 mL of CTAB reagent (2.5 g in 100 mL of 0.2 mol/L NaCl solution) was added to 1 mL of different serial dilutions of standard HA (1 mg/mL) and shaken gently to ensure complete mixing. The mixture was left for 9 min and then the absorbance was read at the 10th minute at 400 nm wavelength using an ultraviolet and visible spectrophotometer (Cary 60 UV-Vis Spectrophotometer, Agilent, Santa Clara, CA, USA). For HA-BSA NPs, the NPs were centrifuged at 13,000 rpm, 4 °C for 20 min (Model 3K-30; Sigma Laborzentrifugen GmbH, Osterode, Germany), and the supernatant was treated as mentioned earlier in the calibration curve. The concentration of free HA was calculated based on the calibration curve of HA and the amount of HA-coated onto the surface of NPs. This was determined by difference using Equation (1):

(1) Amount of HA coat=Total amount of HA added−Free HA in the supernatant

### 2.5. Physicochemical Characterization of Prepared BSA NPs

#### 2.5.1. Measurement of Particle Size, Zeta Potential and Polydispersity Index

The particle size (PS), polydispersity index (PDI), and zeta potential (ZP) were evaluated at 25 °C and angle of 173° using Malvern Zetasizer Nano-ZS, Malvern instrument, (Malvern, UK). The separated BSA NPs and HA-BSA NPs were reconstituted in filtered deionized water before analysis and sonicated for 5 min. After suitable dilutions, measurements were determined in triplicates, and results were represented as mean value ± standard deviation (SD).

#### 2.5.2. Measurement of Percentage Entrapment Efficiency (%EE) and Loading Content (%LC)

Briefly, the *NPs* dispersion was centrifuged at 13,000 rpm, 4 °C for 30 min (Model 3K-30; Sigma Laborzentrifugen GmbH, Osterode, Germany). The concentration of unentrapped free *FLUR* in the supernatant was measured spectrophotometrically at 247 nm using an ultraviolet and visible spectrophotometer (Cary 60 UV-Vis Spectrophotometer, Agilent, Santa Clara, CA, USA). Samples were measured in triplicates and represented as mean value ± SD. %*EE* and %*LC* were calculated using Equations (2) and (3):

(2) $$\%EE=\frac{\left(Total\;FLUR\;concentration-Concentration\;of\;free\;unentrapped\;FLUR\right)}{Total\;FLUR\;concentration}\times 100\;\;\;$$

(3) $$\%\;LC=\frac{Concentartion\;of\;entrapped\;FLUR}{Weight\;of\;NPs}\times 100$$

#### 2.5.3. Transmission Electron Microscopy (TEM)

The morphological examination of FLUR-loaded BSA NPs and FLUR-loaded HA-BSA NPs was investigated using transmission electron microscopy (JEM-1400 Plus 120 kV, Joel, Peabody, MA, USA). A small drop of the sample was placed on a carbon-coated copper grid to adhere the particles on the carbon substrate. Samples were air-dried and examined at 20K× magnification power.

#### 2.5.4. Differential Scanning Calorimetry (DSC)

To investigate the possible interactions and in what form FLUR exists in BSA NPs and HA-BSA NPs, the thermal properties of the prepared NPs were assessed. Samples included BSA, FLUR, HA, and lyophilized [blank BSA NPs (F3), blank HA-BSA NPs (F5), FLUR-loaded BSA NPs (F7), FLUR-loaded HA-BSA NPs (F11)]. Powdered sample (5 mg) was then placed in a sealed aluminum pan and scanned in a differential scanning calorimetric instrument (DSC-60A, SHIMADZU, Kyoto, Japan), with a heating rate of 10 °C/min from 30 to 500 °C under constant purging of nitrogen atmosphere at a flow rate of 20 mL/min. A control empty pan was subjected to the same conditions.

#### 2.5.5. X-ray Diffraction (XRD) Analysis

To further estimate the crystallinity of the FLUR-loaded in BSA NPs and HA-BSA NPs, X-ray diffraction analysis was executed using X-ray diffraction (XRD, BRUKER D2-Phaser; Madison, WI, USA) with a voltage of 30 kV and current of 10 mA. The scanning region of the diffraction angle, 2θ, was 10° to 40° with a step size of 0.02°. Similar to DSC, samples included pure BSA, FLUR, HA, and lyophilized samples of each of the following formulations: FLUR loaded BSA NPs (F7), and FLUR loaded HA-BSA NPs (F11).

#### 2.5.6. In Vitro Release of FLUR from BSA NPs

Release of FLUR from BSA NPs (F7) and HA-BSA NPs (F11) was investigated by dialysis bag diffusion method in phosphate buffer saline (PBS) of pH 7.4. Appropriate volumes of F7 and F11 (equivalent to 5 mg FLUR) were instilled in dialysis bags (molecular cut-off weight of 12–14 kDa) and then immersed in 40 mL phosphate buffer saline (pH 7.4) at 37 ± 0.1 °C and 100 rpm to ensure sink condition. Samples were withdrawn at different time intervals and replaced by the same volume of fresh PBS of pH 7.4. FLUR concentration was then determined using an ultraviolet and visible spectrophotometer at 247 nm. Percentage cumulative drug release was then calculated and corrected then plotted against time. In addition, the release of FLUR from suspension in deionized water was investigated for comparison. The measurements were performed in triplicates and the results were represented as mean values ± SD.

#### 2.5.7. Stability Study

The stability of FLUR-loaded BSA NPs (F7) and FLUR-loaded HA-BSA NPs (F11) was estimated at 4 °C. A suspension of freshly prepared NPs was kept in the refrigerator in well-closed containers at 4 °C and examined for mean PS, ZP, PDI, and %EE after six months.

### 2.6. In Vivo Characterization of the Prepared NPs

#### 2.6.1. Animals

Thirty Adult male Sprague-Dawley rats weighing (200 ± 20 g) were obtained from Medical Research Institute, Alexandria University, Egypt, and maintained under a temperature of 25 ± 1 °C and controlled relative humidity of 50–60% with 12 h dark/night cycle. Rats were acclimatized for one week. Rats were given standard pellets diet and distilled water during the two weeks of the experiment. They were divided into six groups, 5 rats in each group (*n* = 5), where group A represented the healthy group (negative control), group B; the arthritic untreated group (positive control), and the treated groups; group C treated with FLUR-loaded HA-BSA NPs (F11), group D treated with blank HA-BSA NPs (F5), group E treated with FLUR-loaded BSA NPs (F7) and group F treated with blank BSA NPs (F3). All procedures were performed following the regulations of the National Research Council’s Guide for the care and use of laboratory animals and approved by the Ethics Commission of Medical Research, Faculty of Pharmacy, Alexandria University Institute (ALEXU-IACUC AU 062021971108). Animals’ distress was reduced following the internationally accepted principles for laboratory use and care of the International Council of Laboratory Animal Science (ICLAS).

#### 2.6.2. Induction of Arthritis

Adjuvant-induced arthritis model (AIA) was performed by injecting 100 μL of complete Freund’s adjuvant in the right knee of each rat [40]. The left knee was kept uninjected as control. Based on the development of symptoms, treatment was initiated on the seventh day of induction and was extended for two weeks. During the treatment period, rats received an intra-articular injection of a dose equivalent to 2.5 mg/Kg flurbiprofen every other day [15].

#### 2.6.3. Knee Joint Swelling

During the experiment, changes in knee diameter were measured to assess the anti-arthritic effect of the various formulations. The knee joint diameter in rats was measured at different time points (zero-point, seven-days post-induction, one- and two weeks post-treatment) using a calibrated manual caliber. Each time, the average value of two perpendicular diameters of the joint was calculated. The percentage change in knee swelling was also calculated and compared to the control left knee. In addition, percentage improvement in knee swelling was also calculated.

#### 2.6.4. Behavioral Testing (Knee Bend Test)

Movement-induced nociception was estimated in all rats by the Knee-Bend method [41] at different time points (zero point, seven days post-induction, one- and two weeks post-treatment). Every time, the same experimenter conducted the testing blindly. The squeaks and/or struggles in response to five alternate flexions and extensions of the knee joint, done within the physiological limits of knee flexion/extension, were counted in the Knee-Bend test. The following types of reactions to each joint movement were used to score the test: (0) no responses to any type of movement of the knee joint, (0.5) difficulty to full range flexion/extension, (1) difficulty to moderate range flexion/extension, (2) vocalization reaction as a response to moderate flexions or extensions. A full range extension is when the knee joint moves at an angle of 180°, a moderate extension is when the angle is between 120° and 150°, a moderate flexion is when the angle is between 45° and 75°, and a full range flexion is when the knee joint moves at an angle of 180°. The sum of the animal’s reactions was used to determine if the animal was experiencing movement-induced nociception.

#### 2.6.5. Termination and Tissue Collection

Two weeks post-treatment, rats were euthanized via inhalation of gas anesthesia followed by exsanguination where blood samples were collected. Knee joints were then separated from different groups where some were frozen at −80 °C and others were fixed in 10% *w*/*v* formalin solution till further analysis.

#### 2.6.6. Evaluation of C-Reactive Protein (CRP) Level

Blood samples collected from all rats at the end of the experiment (two weeks post-treatment) were centrifuged at 5000 rpm for 10 min at 4 °C, serum was separated and stored at −80 °C for biochemical analysis. Serum samples collected at the end of the experiment were used to evaluate CRP level (LINEAR CHEMICALS S.L, Barcelona, Spain) according to the manufacturer’s instructions.

#### 2.6.7. Measurement of Inflammatory Mediators in Serum

Measurement of inflammatory mediators such as TNF-α and IL-6 was carried out on the collected serum using Enzyme-linked immune sorbent assay (ELISA) using TNF-α and IL-6 ELISA kits, respectively (China Bio-Techne China Co., Ltd., Shanghai, China). All ELISA test kits were used according to the manufacturers’ instructions.

#### 2.6.8. Evaluation of Inflammatory Mediators’ Expression by Western Blotting

The frozen knee articular cartilage tissues were homogenized (100 mg tissue in 1 mL PBS + protease inhibitor). Protein concentrations were quantified using a BCA reagent kit (Pierce Biotechnology, Rockford, IL, USA). Equal volumes (20 µL) of lysate protein were separated on a 12% *w*/*v* SDS-PAGE (Sodium Dodecyl Sulfate Polyacrylamide Gel Electrophoresis). The separated proteins were then transferred to a polyvinylidene fluoride (PVDF) membrane. The membrane was blocked in tris-buffered saline with Tween 20 (TBST) buffer and 3% *w*/*v* bovine serum albumin (BSA) at room temperature for 1 h. Membranes were immersed in Tris-buffered saline with Tween 20 (TBST) and incubated for three hours at room temperature with the primary antibody; IL-6 (Catalogue no: p620, 1:1000 dilution; Thermo Fisher Scientific, Waltham, MA, USA) and TNF-α (Catalogue no: PA5-19810, 1 ug/mL dilution; Thermo Fisher Scientific). Beta Actin primary antibody (Catalogue no: MA1-91399, 1:5000 dilution; Thermo Fisher Scientific, USA) was used as a quantitative control. Membranes were then incubated for one hour with the secondary antibody (Goat anti-rabbit IgG- HRP-lmg Goat mab -Novus Biologicals (Littleton, CO, USA), 1:5000 dilution). The chemiluminescent substrate (Clarity^TM^ Western ECL substrate—BIO-RAD, Hercules, CA, USA cat#170-5060) was used following the manufacturer’s recommendation. Signals were captured using a CCD camera-based imager, and the bands’ intensity of the target proteins against the control sample was quantified using image J analysis software (1.8.0_172, NIH, Stapleton, NY, USA). 

#### 2.6.9. Histopathological Examination

Knee joints fixed in 10% *w*/*v* phosphate-buffered formalin were subsequently decalcified in 5% *w*/*v* formic acid for 72 h. Samples were processed into wax blocks and stained with hematoxylin-eosin and examined microscopically to investigate the anti-arthritic effect of all formulations. 

#### 2.6.10. Immunohistochemistry

The formalin-fixed, paraffin-soaked joint tissues (including synovium and cartilage) were sliced at a thickness of 5 μm. Then, all sections were deparaffinized and rehydrated. Sections were then incubated in 3% *v*/*v* H_2_O_2_ in methanol for 10–15 min at room temperature. On the next day, sections were incubated with the following rabbit polyclonal antibody: (anti-INOS, A0312) (diluted 1:100, ABclonal, Woburn, MA, USA), then washed with PBS four times followed by incubation with peroxidase-compatible chromogen. Dried sections were then stained by freshly prepared DAB (diaminobenzidine) to allow observation under a light microscope. The stained sections were placed on a slice shelf and embedded in distilled water for 5 min and counterstained with Harris stain. The negative controls were performed by substituting the primary antibody with non-immune serum.

### 2.7. Statistical Analysis

Studies were performed at least in triplicates and all data were statistically analyzed using GraphPad Prism 7 software using *t*-test, one-way and two-way analyses of variance (ANOVA) with Tukey’s multiple comparisons test for pairwise comparisons. Values were considered significant when *p* ≤ 0.05.

## 3. Results and Discussion

### 3.1. Optimization and Physicochemical Characterization of the Prepared BSA NPs

The challenge in the preparation of FLUR-loaded BSA NPs is to obtain a formulation with optimum particle size (PS) and optimum percentage entrapment efficiency (%EE). In this study, BSA NPs were prepared by the previously reported modified nano-emulsification method. This method is based on the technique first used by Desai et al. [42] for the production of Abraxane; a human serum albumin-loaded paclitaxel nanoparticulate formulation approved by FDA for the treatment of breast cancer [42]. To optimize the preparation of BSA NPs, the effect of varying amounts of BSA and FLUR on PS was investigated. As shown in Table 1, for blank uncoated formulations, increasing the BSA amount from 30 mg to 50 mg resulted in a significant increase (*p* ≤ 0.05) in mean PS from 349.61 ± 117.92 nm to 506.31 ± 52.12 nm for F1 and F2, respectively. PS significantly decreased (*p* ≤ 0.0001) to 163.83 ± 24.39 nm for F3 when BSA was further increased to 60 mg. Increasing BSA amount was also accompanied by a decrease in PDI values from 0.69 ± 0.09 to 0.58 ± 0.04 and 0.35 ± 0.12 for F1, F2, and F3, respectively. 

Our results were in agreement with work done by Rahimnejad et al. [43] and Radwan et al. [44] who also documented a decrease in PS of BSA NPs upon increasing BSA amount. On the other hand, Galisteo-González and Molina-Bolívar showed contradicting results where an increase in PS values from 75 nm to 135 nm was observed when the BSA amount increased from 12 mg to 100 mg [45]. 

The variation in PS upon changing BSA amount may be attributed to different factors during the preparation of BSA NPs such as homogenization speed, homogenization cycles, and the presence of chloroform that can affect the conformation of BSA NPs [46]. According to our results, F3 prepared using 60 mg BSA was selected for further development owing to its small PS and acceptable PDI values. 

Variation in FLUR loading also showed a significant effect on the PS of uncoated BSA NPs. Increasing the amount of FLUR from 15 to 25 and then to 40 mg resulted in a significant increase in PS (*p* ≤ 0.0001) from 172.21 ± 12.08 nm to 601.63 ± 61.58 nm and 2756 ± 257.92 nm for F7, F8, and F9, respectively. This was also accompanied by a change in PDI values from 0.35 ± 0.02 to 0.59 ± 0.06 and 0.47 ± 0.19 for F7, F8, and F9, respectively. Accordingly, F7 prepared using 15 mg FLUR was selected as the optimum formulation for further studies.

Coating the selected FLUR-loaded BSA NPs (F7) and blank BSA NPs (F3) with HA was performed in an attempt to achieve active targeting to the inflamed joints via selective binding to CD44 receptors which are over-expressed in the inflamed joints [37]. As illustrated in Table 1, increasing the HA amount added to FLUR-loaded BSA NPs dispersion from 1 mg/mL to 2 mg/mL resulted in a significant decrease in PS values (*p* ≤ 0.001) from 531.91 ± 6.58 nm to 257.12 ± 2.54 nm for F10 and F11, respectively. This was followed by a significant increase in PS (*p* ≤ 0.0001) at 4 mg HA to 869.81 ± 128.52 nm for F12. The same pattern was obtained upon coating the blank BSA nanoparticles (F4 to F6). In addition, increasing HA concentration resulted in a change in PDI values from 0.44 ± 0.08 to 0.25 ± 0.01 and 0.98 ± 0.03 for loaded F10, F11, and F12, respectively. It has been reported by Radwan et al. that increasing HA amount from 1 mg to 4 mg resulted in a significant increase in mean PS [44]. Thus, F5 (blank NP) and F11 (FLUR loaded) coated with 2 mg/mL HA were selected for further investigations. 

Regarding ZP, coating the BSA NPs with 2 mg HA resulted in an increase in the net negative charge from −6 ± 1.15 mv for F7 to −48 ± 3 mv for F11 and from −8 ± 1.53 mv to −24 ± 0 mv for F3 and F5, respectively (Table 1). The increase in negative charge was attributed to the adsorption of HA on the surface of albumin nanoparticles which also confirmed the success of coating NPs with HA. The efficiency of the coating was also determined by estimating the amount of HA that coated the NPs. The turbidimetric method was chosen as an efficient and rapid method to determine HA [39]. Amount of turbidity developed when CTAB is added to a HA solution is directly proportional to the amount of HA in the system. The amount of HA adsorbed on the surface of BSA NPs was 64.42 ± 0.02% and 66.34 ± 0.03% for F11 and F5, respectively, which confirmed successful coating of the developed nanoparticles with HA. 

Regarding the percentage entrapment efficiency of FLUR in BSA NPs, the assay of unentrapped free drug in the selected formulations revealed that %EE of FLUR in F7 was 90.35 ± 0.99% with LC of 18.06 ± 0.19% and was 90.12 ± 1.01% with LC of 17.55 ± 0.21% for F11.

#### 3.1.1. Transmission Electron Microscopy (TEM)

**Figure 1:**
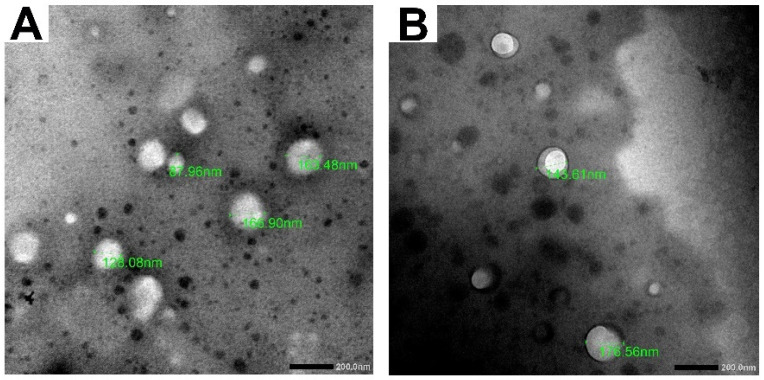
TEM images of (**A**) FLUR loaded BSA NPs and (**B**) FLUR loaded HA-BSA NPs at 20K magnification power.

Morphological examination of FLUR-loaded HA-BSA NPs (F11) and FLUR-loaded BSA NPs (F7) confirmed the formation of spherical particles with homogenous size distribution in both uncoated and HA-coated formulas as shown in Figure 1. In addition, the absence of FLUR crystals in the prepared BSA NPs was revealed by TEM, which is considered solid evidence of high FLUR encapsulation in the NPs.

#### 3.1.2. Differential Scanning Calorimetry (DSC) and X-ray Diffraction (XRD) Analysis

**Figure 2:**
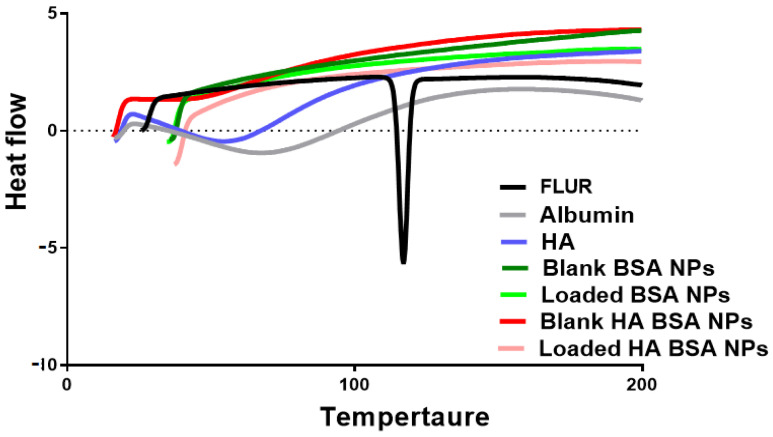
DSC thermograms of FLUR, Albumin (BSA), HA, (Blank BSA NPs (F3), Blank HA-BSA NPs (F5), FLUR loaded BSA NPs (F7) and FLUR loaded HA-BSA NPs (F11).

DSC was utilized to investigate in what form FLUR existed in the prepared NPs. As illustrated in Figure 2, the pure FLUR thermogram showed a sharp endothermic peak at 113.93 °C which corresponds to the melting point of its crystalline form [13,15]. The DSC of pure BSA exhibited a broad endothermic peak at 66.09 °C corresponding to the denaturation peak of BSA [47]. HA thermogram showed a broad endothermic peak at 52.93 °C and this is thought to be associated with the loss of moisture remaining after the drying procedure [48]. The disappearance of peaks of FLUR at 113.93 °C corresponds to the melting point of its crystalline form in FLUR-loaded BSA NPs (F7) and FLUR-loaded HA-BSA NPs (F11) indicating the encapsulation of FLUR in BSA NPs in an amorphous form.

**Figure 3:**
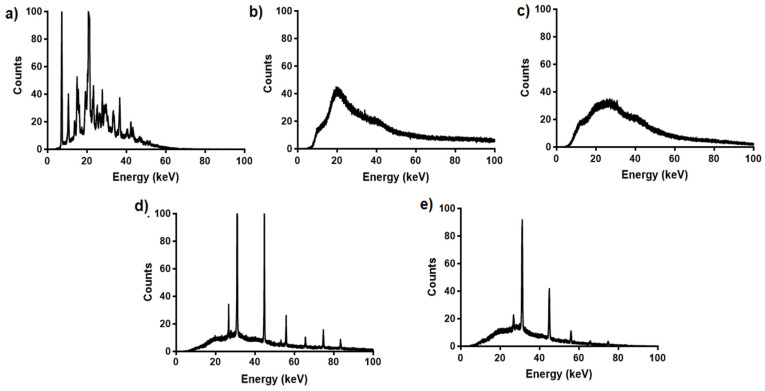
X-ray powder diffraction spectra of (**a**) FLUR, (**b**) BSA, (**c**) HA, (**d**) FLUR loaded BSA NPs (F7) and (**e**) FLUR loaded HA-BSA NPs (F11).

X-ray powder diffraction analysis, Figure 3, confirmed the results obtained by DSC. The XRD spectra show that FLUR exhibited strong sharp peaks corresponding to the crystalline form. However, the intensity of these sharp peaks decreased in both FLUR-loaded BSA NPs (F7) and FLUR-loaded HA-BSA NPs (F11) confirming the existence of the amorphous form of FLUR in the selected formulations. The appearance of certain sharp peaks in drug loaded NPs may be attributed to the non-encapsulated drug present near the surface of NPs.

#### 3.1.3. In Vitro Release of FLUR from BSA NPs

Since the rapid clearance of the drug from joints is a major challenge in the development of intra-articular formulations, formulating therapeutic agents in a controlled release system with high joint retention is an excellent way to design an ideal intra-articular drug delivery platform. [17].

**Figure 4:**
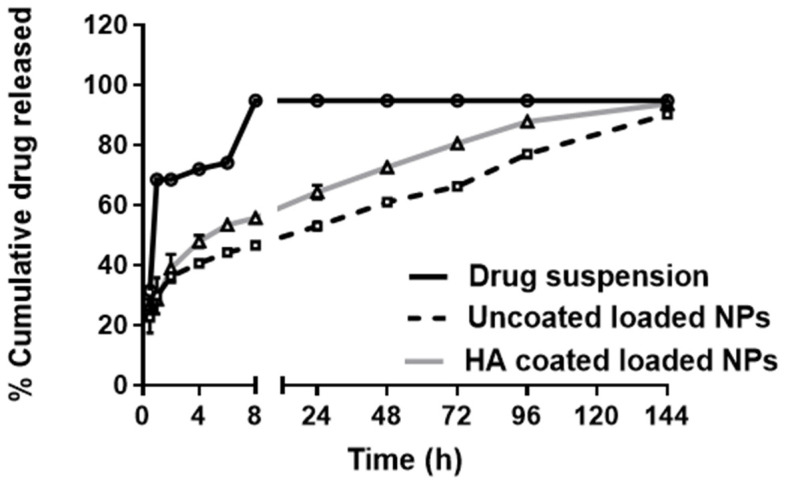
In vitro release of FLUR (5 mg) from FLUR-loaded HA-BSA NPs (F11), FLUR-loaded BSA NPs (F7), and FLUR suspension in PBS 7.4 at 37 ± 1 °C at 100 rpm. Each point represents mean ± SD (*n* = 3).

In vitro release of drug from FLUR-loaded BSA NPs (F7) and FLUR-loaded HA-BSA NPs (F11) was performed to investigate the effect of FLUR encapsulation on its release properties. The prepared formulations were compared to the suspension of free FLUR crystalline powder. As Figure 4 illustrates, the in vitro release profiles for free FLUR suspension showed rapid release of FLUR up to 30 ± 1.05% in the first 30 min. The release then increased up to 68 ± 1.05% within 2 h with a complete release (95 ± 1.05%) after 8 h. On the other hand, FLUR-loaded BSA NPs (F7) and FLUR-loaded HA-BSA NPs (F11) showed a more sustained release pattern. An initial burst effect occurred in the first few hours where a 40 ± 0.03% and 48 ± 0.02% FLUR release was observed in the first 4 h, respectively. This was followed by cumulative FLUR release of 90 ± 0.69% and 93.81 ± 1.17% after 6 days for FLUR-loaded BSA NPs (F7) and FLUR-loaded HA-BSA NPs (F11), consecutively. A sustained-release effect lasting for 144 h was observed which is attributed to the encapsulation of FLUR in NPS. 

The initial burst release effect in the first four hours may be attributed to non-entrapped FLUR present near the surface of NPs. Release of FLUR from FLUR-loaded HA-BSA NPS (F11) was faster compared to FLUR-loaded BSA NPs (F7). This may be due to the presence of sodium hydroxide in FLUR-loaded HA-BSA NPs; used to adjust the pH of the preparation during the coating step which may have caused an increase in the solubility of FLUR [49] thus, affecting its release.

In contrast, Zhou et al. [50] reported the slower release of prednisolone from HA-coated solid lipid nanoparticles than uncoated loaded solid lipid nanoparticles. Comparably, Khan et al. [51] reported that FLUR-loaded pH-sensitive nanoparticles showed a sustained release effect where 30% of FLUR was released from FLUR NPs in the first 30 min followed by 78% of drug release within 12 h at pH 7.4. 

However, Yan et al. [52], who created prednisolone and curcumin-loaded albumin nanoparticles for arthritis therapy, found that they demonstrated a prolonged release pattern as compared to free drug formulation which was similar to our findings. 

#### 3.1.4. Stability Study

The stability of both FLUR-loaded BSA NPs (F7) and FLUR-loaded HA-BSA NPs (F11) was tested after six months of storage at 4 °C regarding PS, PDI, and %EE. PS analysis of FLUR-loaded BSA NPs (F7) showed a significant increase (*p* ≤ 0.01) in mean PS values from 172 ± 12.08 nm at zero time to 737.91 ± 43.01 nm after six months of storage (Table 2). In addition, change in PDI values upon storage from 0.35 ± 0.02 to 0.54 ± 0.03 was also observed with nearly similar zeta potential values during six months, −6 ± 1.15 mv at zero time and −7 ± 0 mv after six months. In addition, %EE of loaded BSA NPs showed significant change (*p* ≤ 0.05) upon storage where %EE was 90.35 ± 0.99% at zero time and 80.17 ± 0.97% after six months.

Results of PS analysis of FLUR-loaded HA-BSA NPs (F11) showed a better stability profile compared to uncoated NPs. Mean PS increased insignificantly (*p* > 0.05) from 257.12 ± 2.54 nm at zero-time storage to 320.52 ± 59.61 nm after six months storage with increase in PDI values from 0.25 ± 0.01 to 0.68 ± 0.06 after six months storage. Zeta potential values retained their high values (−36 ± 0 mv) after six months indicating the stability of HA coating on the surface of BSA NPs during storage for six months. Unfortunately, there was a significant change (*p* ≤ 0.05) in EE% during the storage period from 90.12 ± 1.06% to 79.16 ± 0.76%.

The high zeta potential value (−48 ± 3 mv) of FLUR-loaded HA-BSA NPs (F11) compared to uncoated HA-BSA NPs (F7) could be the reason for their PS stability. Zeta potential is used as a measure of NP stability, and greater ZP values prevent NP aggregation [53]. 

A similar finding was reported, where the stability of HA-coated and uncoated dexamethasone-loaded chitosan nanoparticles was examined after storage at 25 °C for 3 months. Results showed that HA-coated NPs showed higher stability behavior with minor changes in physical properties of coated NPs compared to the uncoated ones [54]. 

### 3.2. In Vivo Characterization of the Prepared NPs

**Figure 5:**
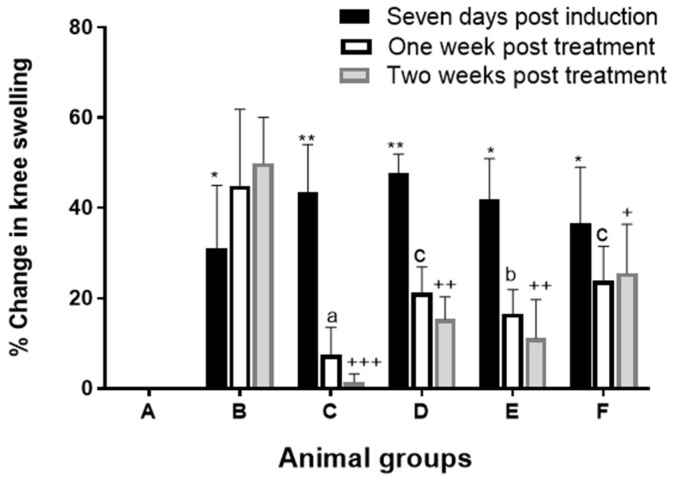
Mean percentage change in knee diameter at different time points (Seven-days post-induction, one- and two-weeks post-treatment for groups (A–F)). where A is the healthy group, B is the untreated arthritic group, C is the group treated with FLUR-loaded HA-BSA NPs, D is the group treated with blank HA-BSA NPs, E is the group treated with FLUR-loaded BSA NPs and F is the group treated with blank BSA NPs. Statistical significance is shown when all groups were compared to healthy group (A) seven-days post-induction (* *p* < 0.001 and ** *p* < 0.0001). All groups were compared to untreated arthritic group (group B) one- week post-treatment with mean values: a < b < c. All groups were also compared to untreated arthritic group (group B) two-weeks post-treatment, with mean values of +++ < ++ < +. Means of similar symbols are statistically insignificant with level of significance *p* ≤ 0.05.

**Figure 6:**
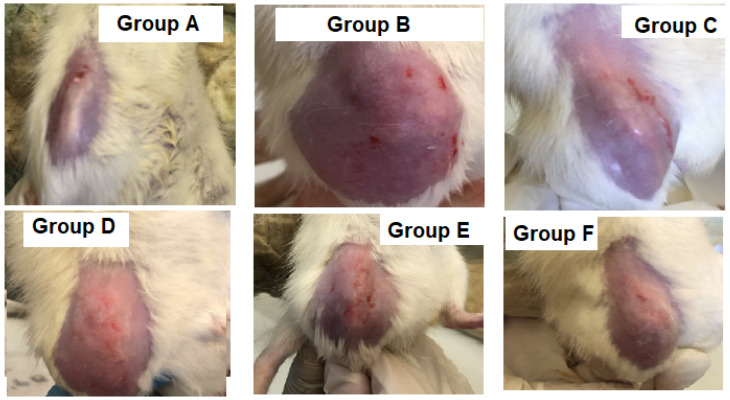
Photographs illustrating knee joints in different groups (**A**–**F**) two weeks after treatment. Each photograph is a representative image of one rat in each group. (**A**) is the healthy group, (**B**) is the untreated arthritic group, (**C**) is the group treated with FLUR-loaded HA-BSA NPs, (**D**) is the group treated with blank HA-BSA NPs, (**E**) is the group treated with FLUR-loaded BSA NPs and (**F**) is the group treated with blank BSA NPs.

Our study aimed at relieving arthritic pain and inflammation via intra-articular administration of FLUR encapsulated in the HA-BSA NPs system. In this work, the adjuvant-induced arthritis model (AIA) was used based on its relevance to physiological changes that take place during inflammatory arthritis progression. This is in addition to being a reliable model for assessing the analgesic and anti-inflammatory properties of drugs [40]. Knee swelling and joint diameter could be observed a few hours after complete Freund’s adjuvant (CFA) injection and tracked throughout the experiment as shown in Figure 5 and Figure 6.

#### 3.2.1. Knee Joint Swelling

Percentage change in knee swelling during the experiment was calculated at different time points (zero-point, seven days post-induction, one week, and two-week post-treatment) as Figure 5 illustrates. Joint diameter increased progressively seven days after AIA induction with maximum swelling and severe erythema (*p* ≤ 0.001) in all arthritic groups as compared to the healthy group (group A).

Significant reduction (*p* ≤ 0.001) in knee swelling was observed one-week post-treatment in group C (7.49 ± 6.08%) (FLUR-loaded HA-BSA NPS; F11) and group E (19.49 ± 7.21%) (FLUR-loaded BSA NPs; F7) when compared to group B (44.94 ± 16.94%) (untreated arthritic rats). Two weeks post-treatment, further reduction in knee swelling was observed. The percentage change in knee swelling decreased significantly (*p* ≤ 0.01) in all treated groups compared to the untreated arthritic group (59.92 ± 24.08%), where rats treated with FLUR-loaded HA-BSA NPs; F11 (group C) showed the highest anti-arthritic effect (1.45 ± 1.79%). 

In addition, percentage improvement in knee swelling was calculated one- and two weeks post-treatment to investigate the anti-arthritic effect of the formulations. One-week post-treatment, FLUR-loaded HA-BSA NPs (F11), and FLUR-loaded BSA NPs (F7) formulations showed 79.68 ± 22.37% and 58.71 ± 17.55% improvement in knee swelling. Moreover, rats treated with blank HA-BSA NPs (F5) (group D) showed significant (*p* ≤ 0.05) improvement in knee swelling (44.08 ± 27.74%) compared to the arthritic untreated group (group B). On the other hand, rats treated with blank BSA NPs (F3) (group F) showed insignificant (*p* > 0.05) improvement in knee swelling compared to untreated arthritic rats (group B), which advocates the anti-arthritic effects of HA. 

Further significant improvement in knee swelling was observed two weeks post-treatment (*p* ≤ 0.001) compared to untreated arthritic group (group B) in the following order: group C (97.13 ± 4.41%) > group E (82.82 ± 14.48%) > group D (69.67 ± 4.31%) > group F (50.63 ± 26.56%).

The above finding confirms the potential anti-arthritic and analgesic effect of FLUR-loaded HA-BSA NPs compared to uncoated BSA NPs and blank treated groups confirming the role of FLUR in reducing inflammation and the role of HA in targeting inflamed joints.

#### 3.2.2. Knee Bend Test

A knee bend test was performed to evaluate the nociception induced by joint movement [41]. The highest score in nociception (around 8) was observed in arthritic animals seven days post-induction of arthritis which is correlated to the increase in knee swelling. As illustrated in Table 3, one-week post-treatment, the measured score decreased to 1.51 ± 1.36 and 2.52 ± 1.76, for group C and group E treated with FLUR-loaded HA-BSA NPs (F11) and FLUR-loaded BSA NPs (F7), respectively, which were statistically different (*p* ≤ 0.001) from the score measured for the untreated arthritic group (group B) (8 ± 2.73).

Blank HA-BSA NPs (group D) and blank BSA NPs (group F) showed significant reduction (*p* ≤ 0.05) in the measured scores, one-week post-treatment to 3.13 ± 1.25 and 4.51 ± 1.25, respectively compared to the untreated group (group B) (8 ± 2.73). Furthermore, two weeks post-treatment, all groups showed a significant (*p* ≤ 0.0001) reduction in the measured score when compared to arthritic untreated rats (group B). The lowest score (1 ± 1.37) was shown by group C which was treated with FLUR-loaded HA-BSA NPs followed by (1.88 ± 1.25) in group E which was treated with FLUR-loaded BSA NPs. Moreover, the drug-free formulas reflected non-negligible effects as they reduced scoring in rats treated with blank HA-BSA NPs (group D) and blank BSA NPs (group F) reflecting the role of BSA and HA in the management of arthritis. From these results, we found that FLUR-loaded HA-BSA NPs exhibited the highest anti-nociceptive effect as compared to other formulations reflecting the role of HA in targeting CD44 receptors in joints and the characteristic anti-inflammatory effect of FLUR in inflamed joints. These results agree with those reported by Li et al. [55] where FLUR-loaded gel and HA decreased knee bend scores in osteoarthritic rats. 

It is worth mentioning that the NSAID diclofenac succeeded in decreasing the nociceptive behavior significantly only one week after injection in the osteoarthritis model by decreasing synovial membrane inflammation, but its efficacy was lost at later time points [41].

#### 3.2.3. Measurement of C-Reactive Protein (CRP) Level

CRP is considered a general biomarker for inflammation or infection [56] where a considerable positive correlation has been observed between serum CRP levels and tissue inflammation scores from synovial biopsy samples in patients with arthritis. 

**Figure 7:**
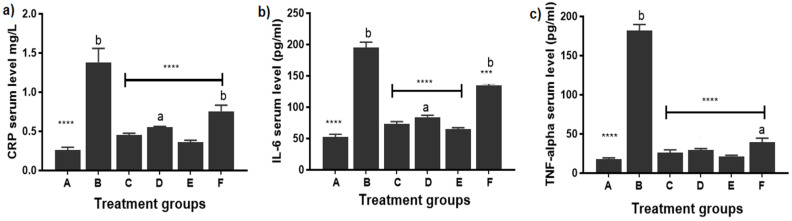
Serum level of (**a**) CRP (mg/L), (**b**) IL-6 (pg/mL) and (**c**) TNF-alpha (pg/mL) in different experimental after treatment. Each point represents the means of 5 replicates. where A is the healthy group, B is the untreated arthritic group, C is the group treated with FLUR-loaded HA-BSA NPs, D is the group treated with blank HA-BSA NPs, E is the group treated with FLUR-loaded BSA NPs and F is the group treated with blank BSA NPs. Statistical significance is shown where **** *p* ≤ 0.0001 and *** *p* ≤ 0.001 when all groups were compared to untreated arthritic rats (group B). All groups were compared to the healthy group (group A) with mean values: a < b; where level of significance was *p* ≤ 0.05.

As depicted in Figure 7a, a significant reduction in CRP levels was observed after treatment with different formulations compared to the arthritic untreated group (*p* ≤ 0.0001). In rats treated with FLUR-loaded HA-BSA NPs (group C) and FLUR-loaded BSA NPs (group E), CRP level was shown to be 0.45 ± 0.03 mg/L (40% reduction) and 0.36 ± 0.03 mg/L (55% reduction) in both groups, respectively, with no significant difference observed between both groups (*p* > 0.05) and no significant difference between them and healthy rat group (group A). Regarding blank formulations, CRP level decreased in groups treated with blank HA-BSA NPS (Group D) to 0.55 ± 0.03 mg/L and in group F which was treated with blank BSA NPs (0.76 ± 0.07 mg/L) but both were still significantly different from group A (healthy rats) (*p* < 0.001). The significant (*p* ≤ 0.01) reduction of CRP level in group C (treated with FLUR-loaded HA-BSA NPS), group D (treated with blank HA-BSA NPS), and group E (treated with FLUR loaded BSA NPS) confirmed the anti-inflammatory effect of both FLUR and HA.

The effect of FLUR on reducing serum CRP level has been reported in a previous study by Esme et al. [57] where the serum CRP level decreased significantly in patients undergoing thoracotomy by adding FLUR to systemic analgesic therapy.

#### 3.2.4. Measurement of Inflammatory Mediators in Serum

IL-6 and TNF-α play crucial roles in the propagation of tissue inflammation and their plasma concentrations which are strongly correlated to the severity of inflammation [58]. Previous studies highlighted the role of both TNF-α and IL-6 as predominant mediators in arthritis and their contribution to the damage of extracellular matrix of bone and cartilage [8,59,60]. As demonstrated in Figure 7b,c, a significant decrease in TNF-α and IL-6 levels (*p* ≤ 0.0001) was shown in all treated groups compared to untreated arthritic rats (group B). Regarding IL-6 levels, rats treated with FLUR-loaded HA-BSA NPs (group C) and FLUR-loaded BSA NPs (group E) showed IL-6 levels of 73.21 ± 3.72 pg/mL and 65.25 ± 2.36 pg/mL in both groups, respectively, with no significant difference observed between both groups and no significant difference to healthy rat group (group A). Additionally, a non-negligible effect in rats treated with blank HA-BSA NPs (group D) and blank BSA (group F) was observed with a significant decrease (*p* ≤ 0.0001) in IL-6 level referring to the role of HA in targeting joints. However, this decrease was still significantly different from healthy rat groups (group A). The significant (*p* ≤ 0.05) reduction of IL-6 level observed by FLUR-loaded HA-BSA NPs and FLUR-loaded BSA NPs formulations compared to blank HA-BSA NPs and blank BSA NPs formulations confirmed the role of FLUR in reducing inflammation.

Concerning TNF-α Level, rats treated with FLUR-loaded HA-BSA NPs (group C) and FLUR-loaded BSA NPs (group E) showed significant (*p* ≤ 0.01) reduction in TNF-α level (26.21 ± 3.52 and 21.52 ± 1.33), respectively, with no significant difference shown between both groups or when compared to the healthy group (group A). In addition, group D (treated with blank HA-BSA NPS) showed a significant reduction in TNF-α level (29.51 ± 1.91 pg/mL). Group F (treated with blank BSA NPs) showed a reduction of 39.62 ± 4.91 pg/mL but was still significantly different from the healthy group (group A). Reduction of serum inflammatory mediators’ level confirmed the anti-arthritic effect of all formulations where FLUR-loaded formulations showed the highest anti-inflammatory effect owing to the effect of FLUR in reducing inflammation. 

It has been reported that different delivery systems such as HA-coated acid-sensitive polymeric nanoparticles loaded with dexamethasone [61], HA-coated solid lipid nanoparticles loaded with prednisolone [50], and HA methotrexate conjugate [34] significantly decreased the level of TNF-α and IL-6 in serum referring to the role of HA in targeting inflamed joints.

In addition, another study reported by Li et al. [55] where the ELISA assay revealed that intra-articular injection of FLUR thermosensitive gel showed a significant reduction in IL-6 levels in joint fluid compared to injection of HA alone reflecting the role of FLUR thermosensitive gel in reducing inflammation.

#### 3.2.5. Evaluation of Inflammatory Mediators’ Expression by Western Blotting

IL-6 and TNF-α are considered the two major pro-inflammatory cytokines that lead to cartilage and bone destruction and are also responsible for the activation of osteoclasts [62]. Understanding the state of cytokines in the synovial fluid after treatment is an approach to assess the anti-inflammatory efficacy of FLUR-loaded NPs on the molecular level. Thus, further examination of their expression in joint tissues was performed at the end of the experiment by western blotting beside their determination in serum by ELISA kits.

**Figure 8:**
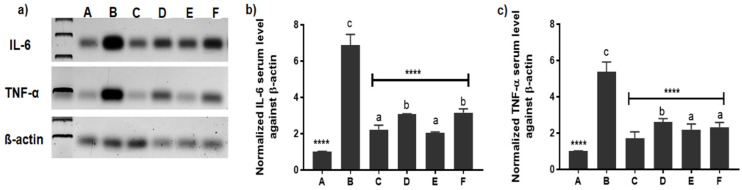
Western blot demonstration of TNF-α and IL-6 expression patterns in the articular cartilages of groups (A–F) 2-week post-treatment. where A is the healthy group, B is the untreated arthritic group, C is the group treated with FLUR-loaded HA-BSA NPs, D is the group treated with blank HA-BSA NPs, E is the group treated with FLUR-loaded BSA NPs and F is the group treated with blank BSA NPs. (**a**) Representative image of western blot showing the expression pattern of IL-6, TNF- α and beta-actin, (**b**) Normalized IL-6 expression in all group samples against beta-actin and (**c**) Normalized TNF-α expression in all group samples against beta-actin. Statistical significance is shown where **** *p* ≤ 0.0001 when all groups were compared to untreated arthritic rats (group B). All groups were also compared to the healthy group (group A) with mean values a < b < c where level of significance was *p* ≤ 0.05.

As illustrated in Figure 8, the results of western blotting showed lower TNF-α and IL-6 levels in healthy animals (group A) but elevated levels were observed in the arthritic untreated group (group B). Reduction of those cytokines levels in all groups was noted after treatment with different formulations (group C, group D, group E, and group F). Our western blotting results demonstrated that the levels of inflammatory factors were lower in the FLUR-loaded HA-BSA NPS group (group C) compared to their levels in the FLUR-loaded BSA NPs (group E) and blank groups (group D and F) confirming the importance of HA in targeting inflamed joints via CD44 receptors. However, all treated groups were significantly different from the healthy rat group (group A) regarding Il-6, while in TNF-α, group C treated with FLUR-loaded HA BSA NPs was the only group that was insignificantly different from group A (healthy rats).

The same findings were also reported previously where HA-coated methotrexate-linked branched polyethyleneimine nanoparticles reduced the TNF-α and IL-6 levels to normal levels after injection [63]. Moreover, hyaluronate gold nanoparticles loaded with tocilizumab complex confirmed the significant reduction of IL-6 level in collagen-induced arthritic mice [64].

Concerning FLUR, it has been reported by Li et al. [55] that expression of TNF-α and IL-6 was decreased in joint tissue after intra-articular injection of HA, FLUR, FLUR free thermosensitive gel, and FLUR thermosensitive gel but a more distinct reduction was observed in FLUR thermosensitive gel treated group. 

#### 3.2.6. Histopathological Examination

**Figure 9:**
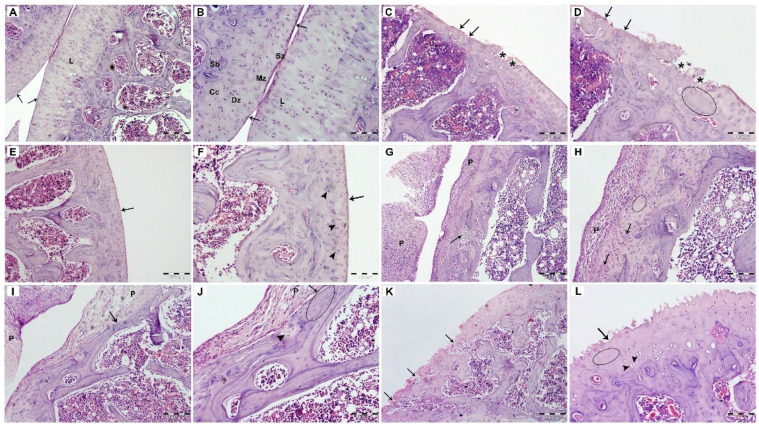
Histopathological examination of joints following H&E staining (H&E stain, Mic. Mag.; (**A**,**C**,**E**,**G**,**I**,**K**) ×100, (**B**,**D**,**F**,**H**,**J**,**L**) ×200). (**A**,**B**) Low and high magnifications of the healthy joints showed different zones of articular cartilage of normal thickness and smooth surface (arrows) including Sz; superficial zone, Mz; mid zone, Dz; deep zone, Cc; calcified cartilage, Sb; subchondral bone, L; scattered chondrocytes within their lacunae. (**C**,**D**) Low and high magnifications of the untreated arthritic group showed irregularity and decreased thickness of the articular cartilage, with hypocellularity (oval shape) and regions of denudation (arrows) and erosions (*). (**E**,**F**) Low and high magnifications of the group treated with FLUR-loaded HA-BSA NPs, revealed more or less normal articular cartilage, with smooth surface (arrows) and nests of chondrocytes (arrowheads). (**G**,**H**) Low and high magnifications of the group treated with blank HA-BSA NPs, revealed that the articular cartilage is covered by the cellular pannus-like tissue (P), Arrows; chondrocytes clusters, oval shape; regions of hypocellularity. (**I**,**J**) Low and high magnifications of the group treated with FLUR-loaded BSA NPs showed decreased thickness of the articular cartilage (arrows), that is covered by highly cellular pannus-like tissue (P), oval shape; areas with low cellularity, arrowhead; low intensity of the matrix. (**K**,**L**) Low and high magnifications of the group treated with blank BSA NPs showed loss of the articular cartilage (arrows), oval shape; hypocellularity and arrowheads; empty lacunae.

The examination of knee joints stained with H& E stain was illustrated in Figure 9A–L. Healthy normal joints showed articular cartilage with normal thickness and smooth surface, while arthritic untreated rat joints showed irregularity and decreased thickness of the articular cartilage, with hypocellularity and extensively regions of erosions. Rats treated by FLUR-loaded HA-BSA NPs, revealed more or less normal articular cartilage, with a smooth surface, increased cellularity, and nests of chondrocytes**.** Treatment with blank HA-coated BSA NPs, resulted in salient improvement in microscopical findings while showing regions of chondrocytes clusters together with regions of hypocellularity. The administration of FLUR-loaded BSA NPs resulted in increased thickness of the articular cartilage while being covered by highly cellular pannus-like tissue. The group treated with the blank BSA NPs showed loss of the articular cartilage and exposure of subchondral bone in some regions. No differences were observed between the healthy rats and those treated with FLUR-loaded HA-coated BSA NPs. Therefore, coating BSA NPs with HA appeared to be safe with the synovium, hence making it suitable for intra-articular delivery. Moreover, blank HA-coated BSA NPs induced a remarkable improvement as compared to the untreated group which emphasizes the reparative actions of HA in inflamed joints. The uptake of HA-BSA NPS by joint cells via the overexpressed CD44 receptors on inflammatory articular cells is confirmed by our histopathology findings, which exactly match the in vivo data.

Previous studies have confirmed that HA-coated NPs accumulate in synovial fluid, allowing for targeted drug delivery, where, HA methotrexate conjugate [34], hyaluronan nanoparticles bearing a γ-secretase inhibitor [35], HA-coated albumin nanoparticles loaded with brucine [37], HA-coated acid-sensitive polymeric nanoparticles loaded with dexamethasone [61], HA-coated methotrexate-linked branched polyethyleneimine nanoparticles [63], and hyaluronate gold nanoparticles complex loaded with tocilizumab [64] appeared to be compatible with the synovium of rats after treatment. 

A similar finding was reported by Kawadkar et al. [15] where intra-articular administration of genipin crosslinked gelatin microspheres of FLUR in knee joints of arthritic rats showed good biocompatibility with the synovium. 

#### 3.2.7. Immunohistochemistry

One of the major inflammatory mediators in arthritis disease is the inducible nitric oxide (iNO) which results in cartilage destruction via various physiological changes during the progression of the disease. As the production of NO in chondrocytes is catalyzed by iNOS, it is involved in the pathophysiology of the disease [65,66]. 

**Figure 10:**
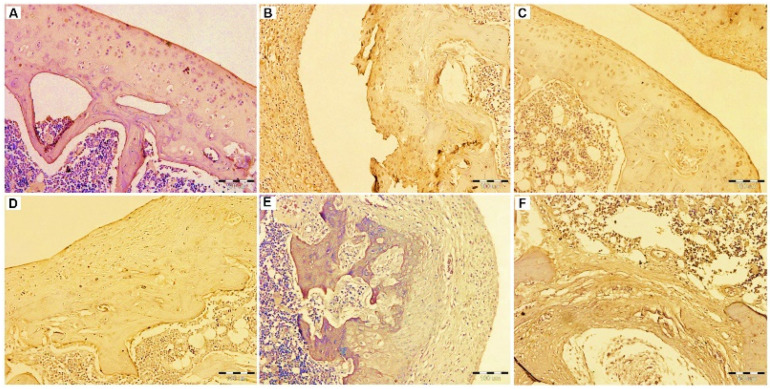
Immuno-histochemical evaluation of the expression of INOS in the knee joints of the rats of the different groups. (**A**) the healthy group. (**B**) the untreated group. (**C**) the group treated by FLUR loaded HA-BSA NPs. (**D**) the group treated by the blank HA-BSA, (**E**) the group treated by FLUR loaded BSA NPs. (**F**) the group treated by blank BSA NPs. [immunostaining, Mic. Mag. ×200].

Herein, immunohistochemical staining iNOS antibody was used to examine its expression level in the different study groups as shown in Figure 10A–F. The arthritic cartilage of the arthritic untreated group showed much higher iNOS expression, with higher staining in the cartilage matrix and chondrocytes. iNOS staining was only weak in the articular cartilage of the healthy control rat. In FLUR-loaded HA-BSA NPs treated group, the level of iNOS expression in the top and middle regions of the articular cartilage was quite low. Intra-articular injection of FLUR-loaded BSA NPs and blank HA-BSA NPS also decreased the level of iNOS expression.

On the other hand, administration of blank BSA NPs was associated with increased iNOS expression in the cartilage matrix. In synovial tissue, coating the FLUR-loaded BSA NPs with HA resulted in the most apparent reduction in iNOS expression. as compared to other treated groups. These results confirmed the role of HA in designing a targeting nano-system of FLUR for the management of arthritis.

The same finding was reported by Gholijania et al. [31] where a significant reduction of NO production in tissue extract was measured after treatment with carvacrol-loaded BSA NPs compared to arthritic rats. In addition, it has been reported that FLUR decreased lipopolysaccharide-induced iNOS expression in RAW 264.7 Macrophages cell culture [67].

## 4. Conclusions

The goal of this research was to develop an intra-articular nanocarrier for an anti-arthritic NSAID (FLUR) to improve its therapeutic outcome. FLUR loaded HA-coated BSA NPs showed an extended release of FLUR in vitro over a period of 6 days. When FLUR-loaded HA-BSA NPs were compared to FLUR-loaded BSA NPs in an arthritis-induced rat model, the anti-arthritic potential of FLUR-loaded HA-BSA NPs was shown to be higher where they showed a significant reduction in knee diameter, inflammatory markers such as IL-6 and TNF-, as well as a drop in iNOS levels in inflamed tissues. These results could be due to the affinity of HA for CD44 receptors at the target location, as well as the non-negligible cartilage healing characteristics of HA. In conclusion, being biomimetic and biocompatible nano-system, HA-coated albumin nanoparticles are considered a promising therapeutic carrier for NSAIDS via active targeting and extending the retention time of drug in inflamed joints after intra-articular injection.

## Figures and Tables

**Table 1:** Optimization and physiochemical characterization of BSA NPs (*n* = 3) showing the effect of BSA, HA, and FLUR concentration on characteristics of BSA NPs.

Formula	Amount (mg)			PS (nm) ± SD	PDI ± SD	ZP (mv) ± SD
	BSA	HA	FLUR			
F1	30	–	–	349.61 ± 117.92	0.69 ± 0.09	−6 ± 1.01
F2	50	–	–	506.31 ± 52.12	0.58 ± 0.04	−6.32 ± 1.15
* F3	60	–	–	163.83 ± 24.39	0.35 ± 0.12	−8 ± 1.53
F4	60	1	–	440.42 ± 40.12	0.59 ± 0.19	−20 ± 0.02
* F5	60	2	–	339.41 ± 19.61	0.23 ± 0.17	−24 ± 0
F6	60	4	–	3046 ± 579.83	0.80 ± 0.28	−24 ± 0.09
* F7	60	–	15	172.21 ± 12.08	0.35 ± 0.02	−6 ± 1.15
F8	60	–	25	601.63 ± 61.58	0.59 ± 0.06	−4.25 ± 2.13
F9	60	–	40	2756 ± 257.92	0.47 ± 0.19	−5.62 ± 3.22
F10	60	1	15	531.91 ± 6.58	0.44 ± 0.08	−27.72 ± 0.35
* F11	60	2	15	257.12 ± 2.54	0.25 ± 0.01	−48 ± 3
F12	60	4	15	869.81 ± 128.52	0.98 ± 0.03	−53 ± 0.23

Abbreviations: PS, particle size; PDI = polydispersity index; ZP = zeta potential; EE, entrapment efficiency; LC = loading content; SD, standard deviation; BSA, bovine serum albumin; FLUR, flurbiprofen; HA, hyaluronic acid. * Is denoted for the chosen optimized formula.

**Table 2:** PS, PDI, ZP, and EE% of freshly prepared NPs and 6-months after storage in the refrigerator at 4 °C (*n* = 3).

Storage Temperature 4 °C				
Formula	Loaded BSA NPs (F7)		Loaded HA-BSA NPs (F11)	
Time (months)	0	6	0	6
PS (nm) ± SD	172 ± 12.08	737.91 ± 43.01	257.12 ± 2.54	320.52 ± 59.61
ZP ± SD	−6 ± 1.15	−7 ± 0	−48 ± 3	−36 ± 0
PDI ± SD	0.35 ± 0.02	0.54 ± 0.03	0.25 ± 0.01	0.68 ± 0.06
EE% ± SD	90.35 ± 0.99	80.17 ± 0.97	90.12 ± 1.06	79.16 ± 0.76

Abbreviations: PS, particle size; PDI, polydispersity index; ZP = zeta potential; EE, entrapment efficiency; SD, standard deviation.

**Table 3:** Behavioral testing (knee bend test), Scoring of the test was measured by the type of reaction to each movement of the joint (*n* = 5).

Group	Zero Time	Seven-Days Post-Induction	One-Week Post-Treatment	Two-Weeks Post-Treatment
Group A	0	0	0	0
Group B	0	8 ± 2.74 ***	8 ± 2.73	10 ± 0
Group C	0	8 ± 2.73 ***	1.51 ± 1.36 ^a^	1 ± 1.37 ^a^
Group D	0	8.75 ± 2.5 ***	3.125 ± 1.25 ^c^	2.52 ± 2.04 ^a^
Group E	0	9 ± 2.23 ***	2.52 ± 1.76 ^b^	1.88 ± 1.25 ^a^
Group F	0	8 ± 2.73 ***	4.51 ± 1.25 ^d^	4 ± 1.37 ^a^

Statistical significance is shown when all groups were compared to healthy group (group A) seven days post-induction (*** *p* ≤ 0.001) and when all groups were compared to untreated arthritic group (group B) one- and two weeks post-treatment, (a < b < c < d) means of similar symbols are statistically insignificant. The level of significance was *p* ≤ 0.05.
